# Calculation of magnetocaloric effect with regard for dependence of heat capacity on magnetic field

**DOI:** 10.1038/s41598-023-37272-0

**Published:** 2023-06-20

**Authors:** Anna Kosogor, Victor A. L’vov

**Affiliations:** 1 Institute of Magnetism NASU and MESU, Kyiv, 03142 Ukraine; 2 Faculty of Physics, University of Vienna, 1090 Vienna, Austria; 3 Taras Shevchenko National University of Kyiv, Kyiv, 01601 Ukraine

**Keywords:** Materials science, Applied physics, Magnetic properties and materials, Phase transitions and critical phenomena

## Abstract

A specific heat of the magnetic solid exhibiting AFM–FM phase transition is computed using the Landau-type theory of phase transitions. The experimentally observed dependence of the specific heat value on the external magnetic field is modelled. It is shown, that this dependence has strong influence on the giant magnetocaloric effect (MCE), which is inherent to the solids exhibiting the phase transitions accompanied by the strong change of magnetization value: the disregard of this dependence leads to the noticeable overestimation of adiabatic temperature change, which is the practically important characteristic of MCE. The temperature change characterizing the giant MCE observed in Fe–Rh alloys is computed. The reasonable agreement between the available experimental data and obtained theoretical results is demonstrated.

## Introduction

Energy efficiency and environmental concerns stimulate the search of new materials for magnetic refrigeration as the alternative to traditional cooling technology^1–3^. The multiferroic materials are considered promising for their use in magnetic refrigerators. These materials exhibit the magnetocaloric effect (MCE), which is defined as the isothermal entropy change or the adiabatic temperature change caused by the increase/decrease of magnetic field $H$ applied to magnetic material. Magnetic refrigeration has the potential to be used at room temperature by employing magnetic refrigerants such as Fe–Rh, Gd_5_Si_2_Ge_2_, Mn-As, Heusler alloys, and many others^4,5^. Moreover, the magnetic refrigeration has been identified as a promising option for hydrogen liquefaction^6^. Some magnetic refrigerator prototypes are already in use^7^.

A giant MCE is associated with the magnetic and magnetostructural phase transitions in solids. The giant magnetocaloric effect in Fe–Rh alloys attracts especial attention because these alloys are among the most promising materials for magnetic refrigeration^8^ and also are attractive for the use in the heat-assisted magnetic recording^9^. The review of experimental studies of giant MCE is presented in Refs.^4,7^. The significant efforts were also made to elaborate theoretical model of this effect^7,10–13^. In particular, a combination of first-principles calculations and Monte Carlo simulations was used for computation of the entropy change from thermodynamic Maxwell relations^10^, the ab initio disordered local moment theory for the first order transition was advanced for the description of MCE^11,12^, the high-throughput computational technique was used to calculate the entropy change related to the electronic, lattice, and magnetic degrees of freedom^13^. To avoid the complicated computations, a simple thermodynamic approach to the description of giant MCE was proposed^14,15^. The approach was based on the minimization of the Gibbs potential and demonstrated quantitative agreement between theoretical and experimental results.

When the giant MCE is studied experimentally, the *direct* measurement of magnetic-field-induced temperature change in the temperature interval of magnetic or magnetostructural phase transition encounters serious difficulties, and therefore, the *indirect experimental technique* of MCE research is widely used (see^1,16^ and references therein). This technique is based on the estimation of temperature change from the experimental temperature dependence of heat capacity, $C_{P} (T)$, or temperature and field dependence of magnetization value, $M(T,H)$. The indirect technique uses the thermodynamic equations interrelating with each other the magnetic-field-induced entropy change, magnetization value and heat capacity. A consistent analysis of the applicability of these equations to the description of giant MCE is presented in Ref.^1^. This analysis is focused on the criticism of MCE evaluation based on thermodynamic Maxwell relations and integral equation for the *field-independent* heat capacity.

In the present communication the theoretical analysis of temperature change caused by giant MCE is carried out with regard for the dependence of heat capacity on the magnetic field value. The analysis is based on the theoretical model which describes phase transition from the low-temperature antiferromagnetic (AFM) phase to the high-temperature ferromagnetic (FM) phase and does not use the thermodynamic Maxwell relations^14,15^.

To prove that this method of problem solution is applicable to the solid exhibiting the phase transition characterized by the considerable change of magnetization value, the quantitative estimate of the temperature change is carried out for Fe–Rh alloy.

Let the magnetic field $H$ be applied to the magnetocaloric material at the *initial temperature *$T$. The evaluation of magnetic-field-induced entropy change is

1 $$\Delta S_{H} \equiv [S(H,T) - S(0,T)],$$

where $S(H,T)$ is specific entropy. However, a practically important characteristic of magnetocaloric material is a magnetic-field-induced temperature change, which is related to the magnetization value $M(T,H)$ and specific heat $C_{P} (T,H)$ of the material as

2 $$\Delta T_{H} = - \int\limits_{0}^{H} {\frac{T}{{C_{P} (T,H)}}\left[ {\frac{dM(T,H)}{{dT}}} \right]} dH,$$

(see e.g.^5^ and references therein). Therefore, the experimental or theoretical dependencies of specific heat on the temperature of magnetocaloric material and on the external magnetic field must be obtained for evaluation of the magnetic-field-induced temperature change. An analysis of $C_{P} (T,H)$ function computed for the alloy exhibiting the AFM–FM phase transition will be performed below for the Fe_49_Rh_51_ alloy, to complete a theoretical description of $\Delta S_{H} (H,T)$ function^15^, by theoretical $\Delta T_{H} (H,T)$ values characterizing the inverse MCE in this alloy.

In the case if the specific heat capacity weakly depends on the magnetic field, the expression

3 $$\Delta S_{H} = \int\limits_{0}^{H} {\frac{dM(T,H)}{{dT}}} dH$$

is widely used to estimate the temperature change from the approximate equality^17–23^

4 $$\Delta T_{H} \approx - \frac{{T\Delta S_{H} }}{{C_{P} (T,H)}}.$$

However, the specific heat capacity of Fe–Rh, Gd, Gd–Si–Ge, Ni–Mn-based Heusler alloys, rare-earth compounds and other materials, which are considered promising for their use in refrigeration technics, noticeably depends on the value of external magnetic field^1,16,24,25^. Doubts about the use of this formula have been expressed previously^1,4,26^, however this approximation is still used^20–23^. The Eq. (4) with $C_{P} (T,H) = const$ is called maximal or highest possible adiabatic temperature change (see e.g.^16^), ignoring the sharp increase of specific heat of Fe–Rh alloy (denominator of the fraction in Eq. (4)) in the phase transition temperature range.

Theoretical estimation of the error caused by the use of approximate equality Eq. (4) instead of Eq. (2) is a primary aim of the present communication. To attain this aim the $C_{P} (T,H)$ function observed in the vicinity of phase transition temperature is modeled using the Landau theory of AFM–FM phase transition.

## Methods

A Landau-type theory describing the temperature- and field-induced AFM–FM phase transitions in Fe_49_Rh_51_ alloy was advanced recently and used for description of inverse MCE^15^. The theory starts from the following expression for magnetic part of Gibbs free energy density of antiferromagnetic solid with two magnetic sublattices:

5 $$G_{m} = \frac{1}{2}J_{0} (T)({\mathbf{M}}_{1}^{2} + {\mathbf{M}}_{2}^{2} ) + J_{12} (T){\mathbf{M}}_{1} {\mathbf{M}}_{2} - ({\mathbf{M}}_{1} + {\mathbf{M}}_{2} ){\mathbf{H}},$$

where ${\mathbf{M}}_{1}$ and ${\mathbf{M}}_{2}$ are the magnetization vectors of magnetic sublattices, the absolute value of these vectors $|{\mathbf{M}}_{1} | = |{\mathbf{M}}_{2} |$ depends on the temperature, ${\mathbf{H}}$ is an external magnetic field, the temperature-dependent parameters $J_{0} (T)$ and $J_{12} (T)$ describe the spin-exchange interaction inside the magnetic sublattices and between them, respectively. Both spin-exchange parameters depend on the volume of antiferromagnet due to the spontaneous and forced magnetostriction (see e.g.^15^). According to Landau theory of phase transitions, the spin-exchange parameters, renormalized by magnetostrictive volume change, depend on the temperature as

6 $$\begin{aligned} & J_{12} (T) = j_{12} (T_{0} - T), \\ & J_{0} (T) = j_{0} (T_{C} - T), \\ \end{aligned}$$

where $T_{0}$ and $T_{C}$ are AFM–FM phase transition temperature and Curie temperature, respectively. The temperature dependence of these parameters results from the fundamental equation $F = U - TS$ interrelating Helmholtz energy $F$ with the internal energy of thermodynamic system $U$ (in this case caused by the spin-exchange process) and the magnetic entropy function $S$, approximated in Landau expansion for the free energy by the second-order terms $j_{0} ({\mathbf{M}}_{1}^{2} + {\mathbf{M}}_{2}^{2} )$ and $j_{12} {\mathbf{M}}_{1} {\mathbf{M}}_{1}$. (The computation of magnetic-field-induced entropy change was performed in Refs.^27,28^ using a Bean–Rodbell theory, which involves the complicated expressions for the magnetic entropy, but $\Delta T_{H}$ value was not calculated).

In the strong magnetic field the magnetization vector ${\mathbf{M}} \equiv {\mathbf{M}}_{1} + {\mathbf{M}}_{2}$ is aligned with vector ${\mathbf{H}}$, and the magnetization value $M(T,H)$ increases in the increasing magnetic field due to the decrease of the angle between the vectors ${\mathbf{M}}_{1}$ and ${\mathbf{M}}_{2}$. When the angle vanishes, the magnetic saturation of AFM phase and the field-induced AFM–FM phase transition take place. Therefore, the saturation value of magnetization of antiferromagnetic phase is equal to magnetization of ferromagnetic phase. Due to this, the free energy densities of ferromagnetic and antiferromagnetic phases in the strong magnetic field can be expressed from Eq. (5) in terms of the magnetization of antiferromagnetic phase $M(T,H)$, and its saturation value $M_{S} (T,H)$ as

7 $$G_{FM} (T,H) = \frac{1}{2}J_{FM} (T)M_{S}^{2} (T,H) - M_{S} (T,H)H,$$

8 $$G_{AFM} (T,H) = \frac{1}{2}J_{AFM} (T)M_{S}^{2} (T,H) - \frac{1}{2}J_{12} (T)M^{2} (T,H),$$

where

9 $$\begin{aligned} & J_{FM} (T) = \frac{1}{2}[J_{0} (T) + J_{12} (T)], \\ & J_{AFM} (T) = J_{FM} (T) - J_{12} (T). \\ \end{aligned}$$

(for more details see^14,15^). The saturation magnetization can be described by the standard function

10 $$M_{S} (T,H) = M(0)\tanh \left[ {\frac{{T_{C} M(T)}}{TM(0)}} \right]{ + }\chi (T)H,$$

where $\chi (T)$ is magnetic susceptibility of the solid in the saturated magnetic states. It should be noted that for the Fe–Rh alloys the Curie temperature $T_{C} \approx 680{\text{ K}}$ is much higher than AFM–FM temperature $T_{0} \approx 310{\text{ K}}$. Due to this the temperature dependence of magnetization is almost constant in the vicinity of AFM–FM phase transition (see e.g.^29^) and can be described, therefore, by Eq. (10). The magnetization of antiferromagnetic phase is expressed as

11 $$M(T,H) = \left\{ {\begin{array}{*{20}l} {HM_{S} (T,H)/H_{AFM - FM} (T)} \hfill & {{\text{if }}H < H_{AFM - FM} (T),} \hfill \\ {M_{S} (T,H)} \hfill & {{\text{otherwise}},} \hfill \\ \end{array} } \right.$$

where

12 $$H_{AFM - FM} (T) = J_{12} (T)M_{S} (T,0)$$

is characteristic field value corresponding to the field-induced AFM–FM phase transition^15^.

The Eqs. (5)–(12) enable the computation of the magnetic parts of the entropy and specific heat from the fundamental thermodynamic equations

13 $$S_{m} = - (\partial G_{m} /\partial T)_{P} ,\quad C_{Pm} = T(\partial S_{m} /\partial T)_{P} .$$

The total heat capacity can be computed then as the sum of the magnetic part and the “vibrational” part, described by Debye theory. The field-induced temperature change, $\Delta T_{H}$, can be computed then in three different ways: first, disregarding the magnetic part of heat capacity, second, from Eq. (2) and third, from Eq. (4). The results of these computations will be compared below, to estimate the error caused by the disregard of magnetic heat capacity or by the use of Eq. (4) instead of Eq. (2).

## Results

To describe the specific heat of Fe–Rh alloys the values of physical parameters involved in Eqs. (5)–(12) have been taken from Ref.^15^. These values are presented in Table 1. These values provided the quantitative description of AFM–FM phase transition experimentally observed in Fe_49_Rh_51_ alloy^29,30^.

**Table 1:** Parameters used for computations.

*T*_0_ (K)	*M*_*S*_ (kA/m)	*j*_12_ (K^–1^)	*j*_0_ (K^–1^)	*T*_*C*_ (K)	*ρ* (kg/m^3^)
310	1350	0.877	3.28	678	10.18 × 10^3^

**Figure 1:**
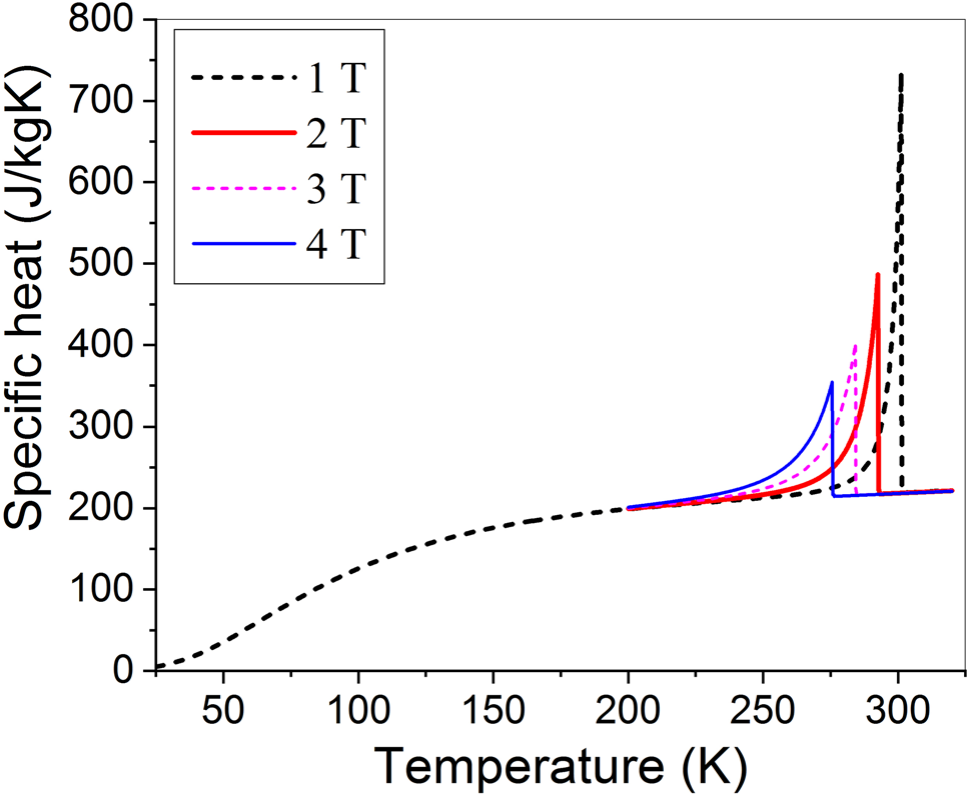
The temperature dependent specific heat computed for the different magnetic field values shown in the Inset.

Figure 1 shows the theoretical temperature dependences of specific heat

14 $$C_{P} (T,H) = C_{Pm} (T,H) + C_{D} (T),$$

where $C_{D}$ is described by the commonly known Debye formula. The specific heat was calculated using the data shown in Table 1 and Debye temperature $T_{D} = 390{\text{ K}}$. (For this value of Debye temperature, the theoretical $C_{P} (T,0)$ curve fits to the experimental one in the temperature range of validity of $C_{P} (T,0) \propto T^{3}$ law^31,32^.)

**Figure 2:**
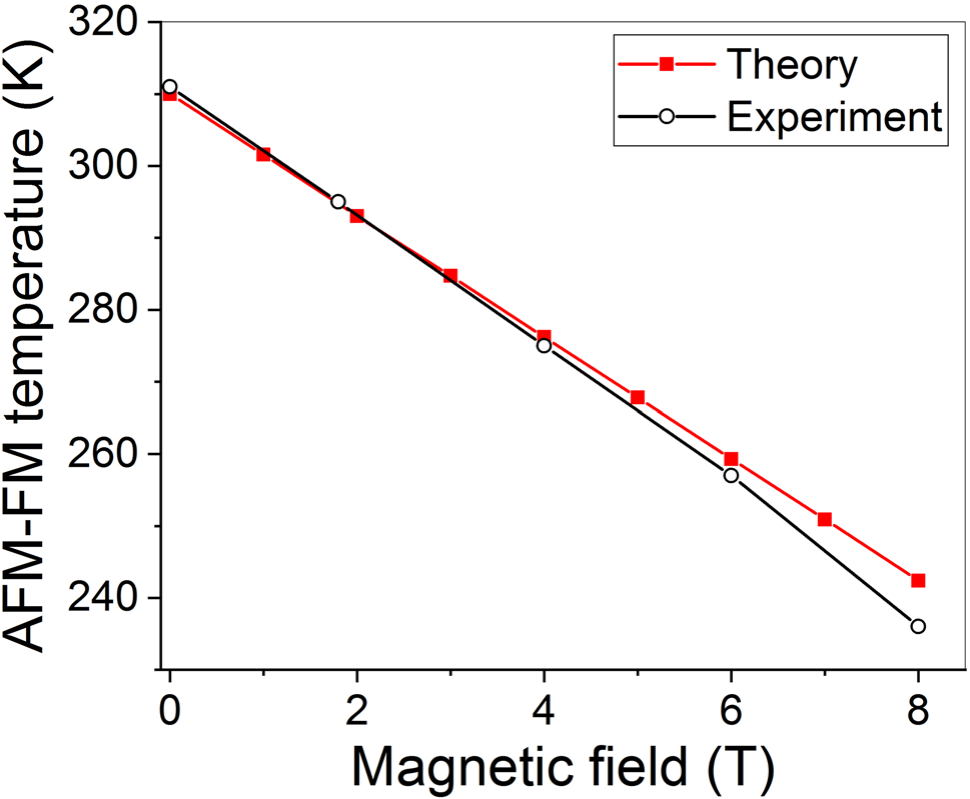
Theoretical and experimental data^33^ of AFM–FM transformation temperatures under different magnetic fields obtained from specific heat peaks.

The influence of external magnetic field on the specific heat of Fe_48_Rh_52_ was studied and the shift of AFM–FM phase transition temperature was observed in Ref.^33^. Figure 2 shows the experimental values of AFM–FM phase transition temperatures, determined from the peaks values of specific heat measured in Ref.^33^, and theoretical values, corresponding to the peaks of the curves depicted in Fig. 1. A good agreement between experimental and theoretical data takes place. The agreement was achieved using only the quantitative characteristics of AFM–FM phase transition derived in Ref^15^ from experimental data reported for Fe_49_Rh_51_^29,30^; no additional fitting parameters were used.

**Figure 3:**
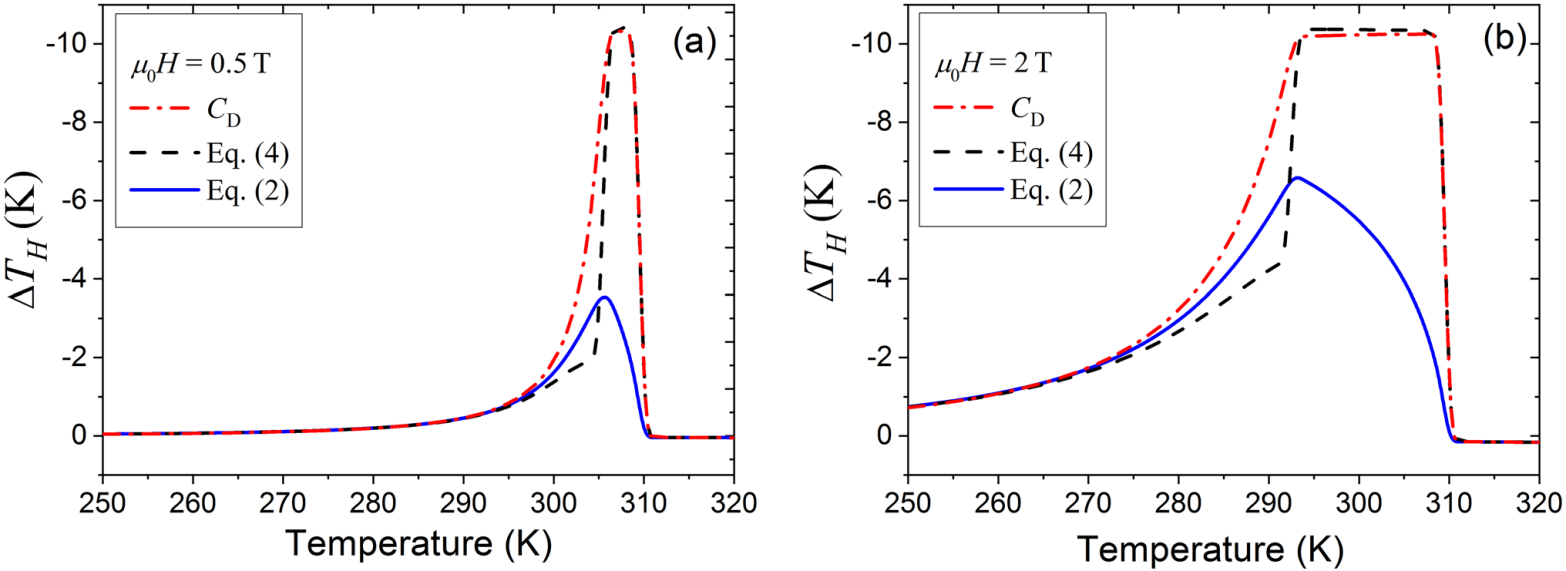
Magnetic-field-induced temperature change computed as a function of initial temperature for different values of applied magnetic field.

The theoretical $C_{P} (T,H)$ curves shown in Fig. 1 have the sharp peaks at the AFM–FM phase transition temperature $T_{0} (H)$. (This temperature is a quasilinear decreasing function of magnetic field^15,29,30^.) Both the height and width of the peak strongly depend on the field value. Therefore, $C_{P} (T,H)$ cannot be excluded from the integration in Eq. (2), and this equation cannot be simplified to Eq. (4). As a consequence, Eq. (4) cannot be used for evaluation of MCE. This fact is illustrated by Fig. 3.

Figure 3a,b shows the magnetic-field-induced temperature change computed in three different ways:

1. from Eq. (4) disregarding the magnetic part of specific heat, i.e., using the value $C_{D} (T)$ resulting from the Debye theory (see dash-doted lines in the figure);
2. from Eq. (4) taking into account the magnetic part of specific heat, i.e., using the value $C_{P} (T,H)$ expressed by Eq. (14) (see dashed lines in the figure);
3. from Eq. (2) taking into account the magnetic part of specific heat (see solid lines in the figure).

**Figure 4:**
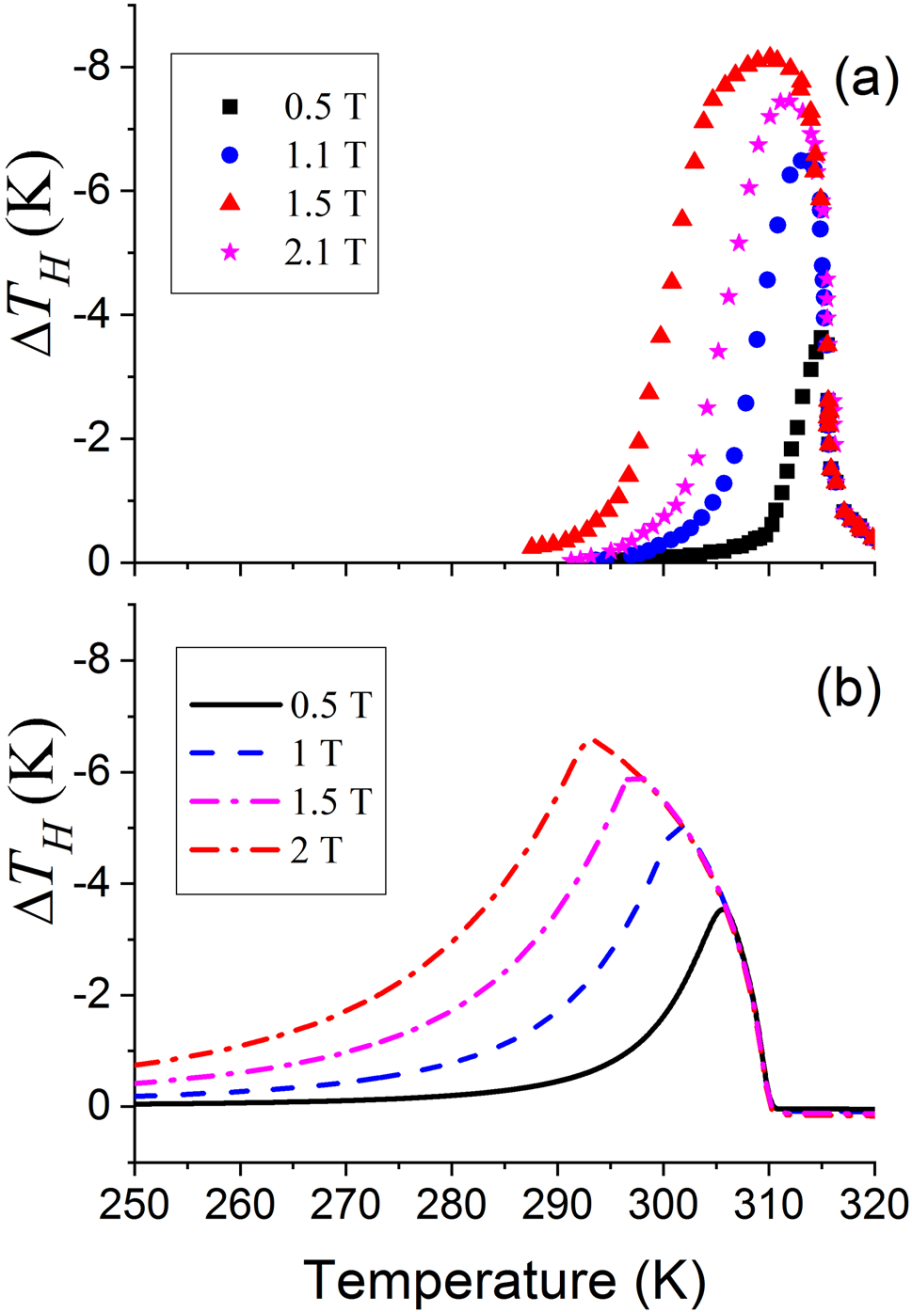
Magnetic-field-induced temperature change measured^19^, (**a**), and computed from Eq. (2), (**b**), for Fe_49_Rh_51_ alloy in different magnetic fields.

A comparison of the curves shown in Fig. 3. by the solid, dashed and dash-doted lines shows that Eq. (4) cannot be used for the evaluation of magnetic-field-induced temperature change if the height and width of $C_{P} (T)$ peaks are noticeably different for different values of the magnetic field. This statement is confirmed by the graphs presented in Fig. 4. Figure 4 shows the resemblance between theoretical temperature change computed from Eq. (2) and experimental values determined in Ref. ^19^. Also Fig. 4 shows that the maximum values of temperature change calculated from Eq. (2) and the corresponding experimental values obtained in Ref. ^19^ strongly depend on the value of applied magnetic field, while the values calculated from Eq. (4) using the Debye formula or Eq. (14) for the total specific heat (see dashed and dash-doted lines in Fig. 3) are almost constant, at variance with experimental data.

## Conclusion

The theoretical consideration of inverse magnetocaloric effect in the solid exhibiting AFM–FM phase transition leads to conclusion that the magnetic-field-induced temperature change, $\Delta T_{H}$, can be evaluated properly from integral relationship (Eq. (2)), while its simplification to the form Eq. (4) can lead to the drastic overestimation of adiabatic temperature change. The discovered above drastic disagreement between the theoretical results, obtained from Eq. (4), and experimental data is caused by the noticeable dependence of the heat capacity of solid, $C_{P} (T,H)$, on the external magnetic field. (This dependence is observed experimentally in the vicinity of AFM–FM phase transition^16,24,33^). It should be noted, that the discovered above drastic disagreement between the theoretical results obtained from Eq. (4) and experimental data hypothetically can be diminished by the proper modeling of experimental dependencies of magnetization value on the temperature and magnetic field, but this disagreement cannot be estimated without use of Eq. (2). It can be concluded, therefore, that if the height and/or width of the peak at the temperature dependence of heat capacity noticeably depend on the value of external magnetic field, the magnetically induced temperature change characterizing MCE can be evaluated from the integral relationship Eq. (2), while the simplified Eq. (4) can give the erroneous result.

## Data Availability

The datasets used and/or analysed during the current study available from the corresponding author on reasonable request.
